# Wildlife in the cloud: A new approach for engaging stakeholders in wildlife management

**DOI:** 10.1007/s13280-015-0706-0

**Published:** 2015-10-27

**Authors:** Guillaume Chapron

**Affiliations:** Grimsö Wildlife Research Station, Swedish University of Agricultural Sciences, 73091 Riddarhyttan, Sweden

**Keywords:** Population models, Cloud-computing, Wildlife management, Moose, Wolf, Bear

## Abstract

Research in wildlife management increasingly relies on quantitative population models. However, a remaining challenge is to have end-users, who are often alienated by mathematics, benefiting from this research. I propose a new approach, ‘wildlife in the cloud,’ to enable active learning by practitioners from cloud-based ecological models whose complexity remains invisible to the user. I argue that this concept carries the potential to overcome limitations of desktop-based software and allows new understandings of human-wildlife systems. This concept is illustrated by presenting an online decision-support tool for moose management in areas with predators in Sweden. The tool takes the form of a user-friendly cloud-app through which users can compare the effects of alternative management decisions, and may feed into adjustment of their hunting strategy. I explain how the dynamic nature of cloud-apps opens the door to different ways of learning, informed by ecological models that can benefit both users and researchers.

## Introduction

Models are recognized as being a valuable tool for sustainably managing wildlife populations (Chapron and Arlettaz 2006; McLane et al. 2011; Schaub and Kéry 2012). Models allow us to mechanistically understand the dynamics of populations, make predictions, and test the possible impact of alternative management strategies (Fryxell et al. 2014). The relevance of models is also substantial when the aim is to establish sustainable hunting or culling quotas (Boyce et al. 2012). The importance of models is not only stressed by academics but also by national, regional, or even local managers increasingly asking for decision-making advice derived from ecological models (Swedish Environmental Protection Agency 2014). However, models have become increasingly complex, up to a point where managers can hardly use them (McNie 2007; Knight et al. 2008). Although applied ecology journals focus on research questions that have a direct relevance to real-world questions (Hulme 2014), the dissemination of model results to end-users remains poor. An increasing number of academic journals require authors to publish the source code of their models and their data, but while this approach is suitable for communication within the research community it leaves wildlife managers facing a very steep learning curve to adjust results to their own situation. As a result, the gap between model-based quantitative research and implementation persists or grows wider (Arlettaz et al. 2010), and the only scientific information that managers can practically handle for making real-world decisions is restricted to expert advice, available meta-analyses, systematic reviews, and compendiums (see e.g., Eycott et al. 2012; Williams et al. 2012; Ojanen et al. 2014). While this information can be very useful, and may be written for an applied audience, it remains static and does not offer the possibility for adaptive decision-making (Walters 1986). One could argue that expert advice is a most fitting way to disseminate research insights to end-users. However, Burgman et al. (2011) found that qualifications, track records, and experience were often poor guides to the performance of scientific experts, leaving open the question of whether model-supported advice may be more reliable than expert advice. This situation is unfortunate as two recently published papers found that managers did change their practice when provided with the relevant information (Dicks et al. 2014; Walsh et al. 2014). There is an urgent need for innovative and quality-insured ways to deliver scientific understanding to end-users (Memmott et al. 2010; Milner-Gulland et al. 2012) and this need of accessible and reliable information is exacerbated in a contemporary focus to avoid unsustainable exploitations of natural resources by humans (Dirzo et al. 2014).

## A new approach

I propose a new approach termed ‘wildlife in the cloud’ to exchange knowledge with end-users and co-develop new ways of learning, thereby arguing that it carries the potential to overcome the limitations described above and allows for new understandings of human-wildlife systems. This approach is based on the idea of ‘cloud-computing’ where only the user interface runs on the user’s machine and the main software runs on a distant server. This approach is becoming ubiquitous in the computer industry (e.g., Microsoft Office 365) but ecological sciences have so far not embraced it. Cloud-computing has the potential of making advanced ecological models widely accessible to the general public. In particular, cloud-computing can address numerous issues that have precluded a wider use of models by professional wildlife managers and groups interested in wildlife management, such as a local hunting association deciding their ground’s quota. Contrary to more traditional desktop-based software (e.g., the most widely used population viability analysis software VORTEX—Lacy 1993), cloud-computing software has its user interface in the form of an app which runs within a web browser (i.e., the browser window is the space within which the app runs, as the desktop is the space within which standard software run) and does not require installation (except loading the webpage). Because web browsers are a central piece of software on modern operating systems, almost any connected device can use a cloud-based app, without installation hassle or compatibility issues. The main software tasks are run on the server side and thus can rely on the processing power of dedicated simulation servers without the user’s own device forming a performance bottleneck. The software can also benefit from optimized numerical libraries (i.e., sets of functions optimized to perform intensive numerical calculations), which would not necessarily be compatible with the user device (e.g., laptops, mobile phones, tablets). This means that users are able to make decisions based on the exact same models as those validated by the scientific community (through e.g., a peer-review publication process). The typically fast computational speed on the server will greatly improve the user experience and means that a cloud-app will run as quickly on a tablet as on a personal computer or a smartphone. Another advantage is that users always have the latest version of the software. If a bug is found, there is no need to contact all users and ask them to update their software, nor is there a need to deal with users who receive different results because of different software versions. The corrected version is simply put on the server and any user loading the cloud-app will then use the most recent version.

## Proof of concept

**Fig. 1:**
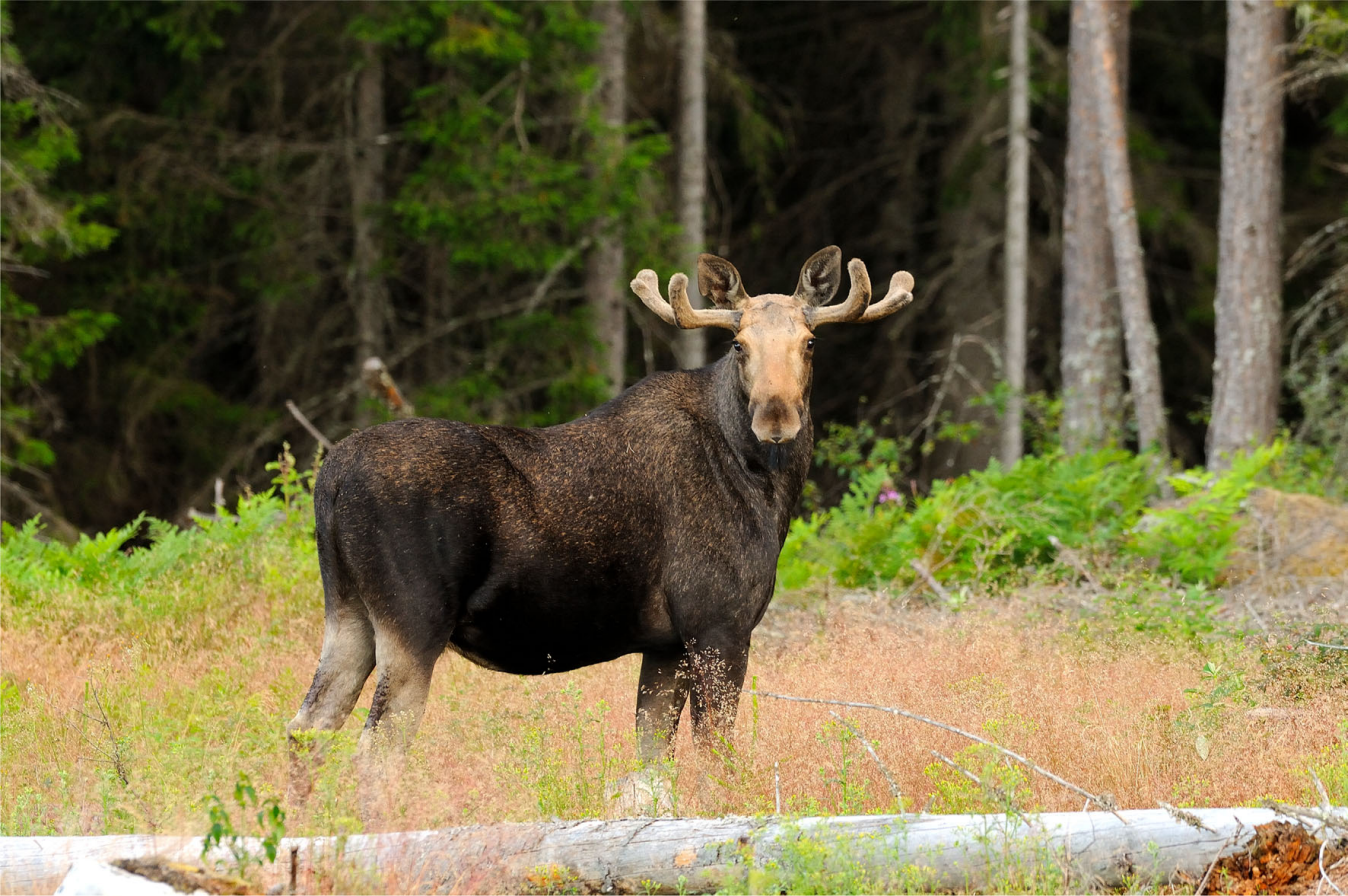
A male moose in one of Sweden’s hunting districts. Moose hunting is an important source of revenue and meat supply to landowners and has also a high recreational value. Photo by Johan Månsson

To illustrate how a cloud-based app for wildlife management could look, I here present a prototype. In 2010, I developed a cloud-app that allowed managers to understand how moose hunting quotas in Sweden would need to be adjusted when large predators are present. Large predators have been recovering in Sweden during the past decades (Chapron et al. 2014). This recovery has had consequences for their main prey, moose, and other ungulates, which need to be understood in order to make sure hunting remains sustainable (Wikenros et al. 2015). The cloud-app was made available in both Swedish and English,^1^ and an online user manual (in Swedish only) provided explanation of the model and parameter settings.^2^ The rationale behind this cloud-app was a bill by the Swedish Government 2009/10:239 (Swedish Government 2009) to reorganize moose hunting—a popular activity in Sweden (Fig. 1). The bill modified the organization of hunting with the purpose of providing ecosystem-based local moose management (Sandström et al. 2013). In this context, the Swedish government gave an assignment to the Swedish University of Agricultural Sciences to develop training material for wildlife management delegations and moose hunters in general. We published the scientific description of a deterministic moose–predator–hunter population model in the journal *Ecological Modelling* (Jonzén et al. 2013) in which we explained how moose harvest strategies needed to be adjusted in the presence of large predators. However, neither this scientific publication nor the associated report aimed for a broader audience (Sand et al. 2011), or assisted managers and hunters in dynamically understanding how moose hunting would need to be adjusted when predators recover in their area. Using the population model (Jonzén et al. 2013) would require people to download command line-based software (R statistical package with additional libraries—R Core Team 2013), familiarity with the programming language (i.e., variable assignment in R), and some understanding of matrix algebra. One cannot expect users to allocate time and efforts to use a tool that was created by researchers for researchers.

**Fig. 2:**
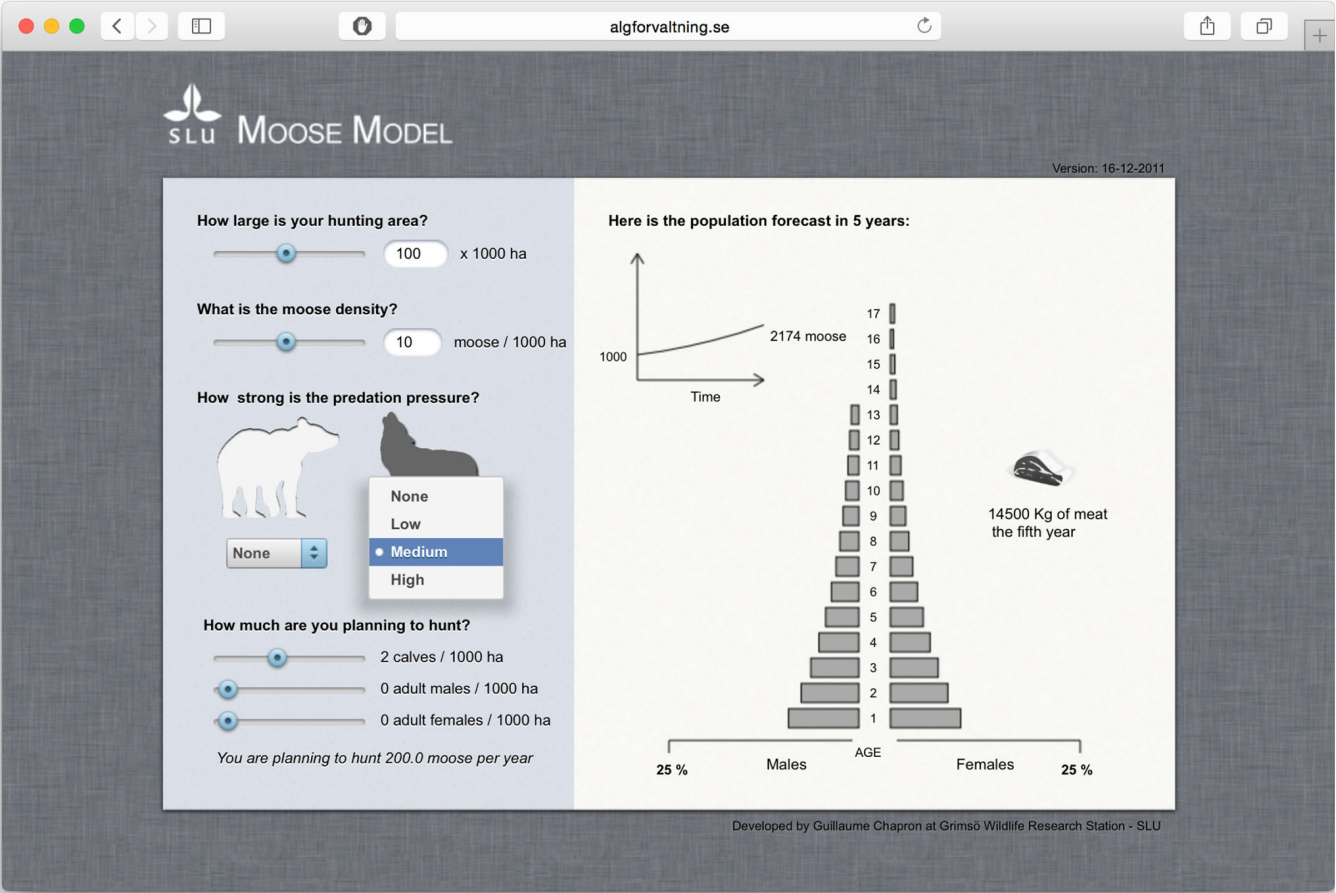
Moose management cloud-app that serves as an interface to a population model running on a distant server. The *left panel* allows the user to set parameters through intuitive controls and the *right panel* instantaneously provides meaningful information from simulation results

To address the range of aspects that prevented a wider use of the model, I designed a cloud-app that allows users to select the size of a hunting area, moose density, qualitative indices of predation pressure, and a planned hunting strategy (see left side of the screenshot in Fig. 2). Moose population simulations are run on a server and sent back to the user’s browser. Displayed results include the 5-year predicted local population trend, the expected yield in the last year, and the asymptotic sex and age structure of the moose population (see right side of the screenshot in Fig. 2). In terms of software architecture, the cloud-app consists of three independent but inter-communicating parts: a user interface written in the language Cappuccino (Cappuccino Project 2013) that is loaded in a browser, a compiled simulation model that runs on a server, and a script (written in a scripted language PHP) that links both interface and the model. When developing software, the interface should be at the core of the user experience (Tidwell 2010), and should ideally be intuitive enough so that it is not seen as an obstacle. In the cloud-app, users can set the exact values of parameters they are likely to know (such as the size of their hunting area) by entering values in text fields or generating those by moving sliders (Fig. 2). The model does not show units from the international system (SI, animals per km^2^) because Swedish wildlife managers and hunters are used to think in thousands of hectares. Some variables for which users are unlikely to know the exact value (such as predator pressure) are set qualitatively with a discrete drop-down menu and visual feedback is given by predator silhouettes with increasing shades of gray for increasing densities of predators. The hunting quota is chosen by mimicking the way hunters distribute their quota, i.e., between calves, adult females, and males. This interface is linked to a sex- and age-structured deterministic matrix model (Jonzén et al. 2013) with 17 age classes for females and 13 age classes for males and additional matrix algebra to include levels of moose predation and hunting. The model is written in the programming language C and compiled with Clang options to optimize computational speed (Clang Team 2014). The interface retrieves results from the model and presents those in an intuitively understandable way, which allows users to focus on what they want to learn. Of particular interest to hunters is the age structure of the moose population to maximize annual meat yield, which is shown in the middle of the results panel and based on empirical body mass data of moose for each sex and age class. Additional visual information is provided by a dynamically changing size of the picture showing the amount of meat available. The use of C libraries for mathematical functions allows for model optimizations and provides a smooth user experience. Each simulation takes less than 1/10th of a second and as a result there is virtually no waiting time from one simulation to the next. This makes it possible to avoid having a ‘run’ button and thus dramatically improve the user experience: by allowing users to seamlessly change parameters and directly observe how this would affect the moose population.

In this cloud-app the interface is clearly focused on numerical values of the moose population structure, but because developing a cloud-app interface is no different from developing a traditional desktop interface, ecological models with detailed graphs or maps outputs could also have been included. Because this cloud-app was created as a proof of concept, it was never advertised to its potential end-users (typically Swedish hunters). Nonetheless, with more than 1 million simulations run, by visitors from across Sweden and other European countries, it has proved to be a popular tool.

## Potential use in wildlife management

The dynamic nature of cloud-apps opens the door to different ways of learning, informed by ecological models that can benefit both users and researchers. I discuss three dimensions of the potential benefits of cloud-apps for wildlife management and wider biodiversity conservation.

### Efficient computing for effective uptake by end-users

The most immediate advantage of cloud-apps is that users can replicate the analysis done by researchers and adjust it to their own context without any programming or quantitative skills or access to powerful computing facilities. Because computations are run on a distant server, an individual user will not be forced to use a less complex, and thus possibly less relevant, model suitable only for personal desktop computers. The moose model would not have been as effective if ran directly on a desktop computer, as simulation time would be considerably greater. This technical complexity barrier is well illustrated by the example of a decision-support system to help wildlife managers and stalkers predict red deer (*Cervus elaphus*) terrain use in Scotland (HillDeer and DeerMap). While effectively integrating deer population and habitat models (see Tremblay et al. 2004), and predicting deer habitat suitability well (particularly when integrating scientific and local knowledge, see Irvine et al. 2009), neither tool has been adopted by estates for use in real life (Maffey et al. 2013). One reason, also identified for other decision-support systems (Uran and Janssen 2003), may have been that the software lacked an intuitive and easy solution to handle a multi-parameter system and user-friendly interface.

### Collective knowledge production and new stakeholder interaction

The opportunity of using advanced models with an intuitive interface also opens possibilities for the collective creation of knowledge by crowdsourcing questions to the interested public. In this context, researchers could, for example, provide a cloud-app and ask users to explore and select management strategies which they think are most appropriate, with the possibility to ‘like’ or up-rank strategies and have the most relevant ones emerging from this exercise. The rationale behind this approach is that local users hold much knowledge about the ecological system they interact with on a regular basis (Irvine et al. 2009). They could thus propose different management strategies (e.g., such as the allocation of a hunting quota among age classes) that otherwise might remain undisclosed to researchers. The approach could be extended to genuine co-creation, with both practitioners and modelers contributing and thereby informing environmental decision-making (Wood et al. 2015). A different collective opportunity potentially brought by a cloud-app could lie in allowing users to interact with their neighbors. For example, further development of the moose cloud-app could include the consideration of what is happening in hunting areas around the selected one and possible population co-dynamics requiring cooperation between users (see also Austin et al. 2013 on the importance of incentivizing collaborative management).

### Understanding usage and users

Learning about the human dimension of wildlife management could also be developed with researchers studying the users of a cloud-app. With the required ethical permits and agreement from users to process individual data, it would be possible to program the cloud-app such that all connection logs are recorded and to extract information from them. Connection logs can contain the IP-address, the time of the connection, and the values of all parameters user has selected for a simulation. Because IP-addresses can be geo-coded, the cloud-app would allow for the development of a spatially explicit understanding of socio-ecological systems. For example, with the moose cloud-app it could be possible to estimate the spatial distribution of predator densities of interest (e.g., in which regions do users choose more often high bear or wolf densities?) or of hunting strategies (e.g., in which regions do users choose more often high calf quotas?).

Understanding how the users interact with the cloud-app can even go one step further. For example, one can analyze which parameters users are more interested in adjusting to reach a particular goal or estimate how long users spent looking at particular kinds of simulations. By designing the cloud-app, so that users are asked to make particular choices under different situations, it would be possible to conduct experimental studies on how people react to particular wildlife management strategies, in a kind of large-scale psychology lab. A cloud-app could also serve as a real-time opinion poll where the reception by stakeholders of management decisions could be tested in advance of these being implemented or even announced.

## Discussion and conclusion

Current barriers to embracing the use of cloud-apps in wildlife management are more conceptual than technical. Broadband internet connection is now widely available across large parts of the countryside of the developed world. Tools to develop adequate cloud-app user interfaces have improved in recent years. As of yet, challenges lie more in the architecture and design of cloud-apps. Ecologists and other researchers may not always have the skills to develop cloud-apps, but they could benefit from working with professional programmers. Alternatively, relatively simple programming frameworks are now widely available. For the moose cloud-app, I used Cappuccino (Cappuccino Project 2013) and developing the app was a hybrid approach between coding a desktop-based software and a website. The growing adoption of the scripted statistical language R has made ecological researchers much more familiar with programming, which likely lowers the knowledge threshold required to develop a cloud-app. In that regard, the new framework Shiny^3^ that allows building interactive reports and visualizations using R could be a simple solution to increase development and use of cloud-apps.

Other challenges—e.g., which meaningful parameter choice should be given to users or how to interpret interest by a particular public and adjust management accordingly—may require collaboration with researchers in sociology, applied psychology, and human–computer interactions (Arts et al. 2015). Finally, making a model available for public use adds a second level of review, this time not by peers but by the members of the public. This will likely increase the quality of models because users will try to understand and scrutinize results and put them into a real-world context. The threshold for ‘acceptance’ in a public-review process may actually be somewhat higher than with an academic peer review and this may force ecological researchers to allocate extra efforts in model validation and quality control.

In conclusion, I encourage applied researchers in ecology who have used simulation models in their work to consider whether cloud-apps could help them to better deliver their results to the public, to provide new ways of understanding their socio-ecological system of interest and to open the door to different ways of learning and practicing natural resource management which can benefit both users and researchers.

## Footnotes

1 www.algforvaltning.se/.

2 http://www.slu.se/sv/centrumbildningar-och-projekt/algforvaltning/manualer/beskattningsstrategier/interaktiv-algforvaltningsmodell/.

3 http://shiny.rstudio.com.
